# The Gut Microbiota Influenced by the Intake of Probiotics and Functional Foods with Prebiotics Can Sustain Wellness and Alleviate Certain Ailments like Gut-Inflammation and Colon-Cancer

**DOI:** 10.3390/microorganisms10030665

**Published:** 2022-03-20

**Authors:** Divakar Dahiya, Poonam Singh Nigam

**Affiliations:** 1 Wexham Park Hospital, Wexham Street, Slough SL2 4HL, Berkshire, UK; ddahiya@hotmail.co.uk; 2 Biomedical Sciences Research Institute, Ulster University, Coleraine BT52 1SA, Northern Ireland, UK

**Keywords:** probiotics, lactic acid bacteria, intestinal microbiota, exopolysaccharide, inflammation, diabetes, antibiotics

## Abstract

The gut microbiota is composed of several microbial strains, with diverse and variable combinations in healthy and sick persons, changing at different stages of life. A healthy balance between host and gut microorganisms must be maintained in order to perform the normal physiological, metabolic, and immune functions and prevent disease development. Disturbances in the balance of the gut microbiota by diverse reasons initiate several health issues and promote the progression of certain diseases. This review is based on published research and reports that describe the role of probiotic microorganisms in the sustainability of health and the alleviation of certain diseases. Information is presented on the GRAS strains that are used as probiotics in the food industry for the production of fermented milk, yogurt, fermented food, functional foods, and probiotic drinks. To maintain a healthy microbiota, probiotic supplements in the form of freeze-dried live cells of probiotic strains are also available in different forms to consumers. The health benefits of lactic acid bacteria and other microorganisms and their role in the control of certain diseases such as gut inflammation, diabetes, and bowel cancer and in the safeguarding of the gut epithelial permeability from the invasion of pathogens are discussed.

## 1. Introduction

Gut microbiota or gut microbiome is a collective term for those microorganisms that live in all vertebrates’ gastrointestinal tract (GIT). In humans, the gut is the main site for the survival of the human microbiota. The microbiota in the gut consists of several strains of bacteria and yeasts. With its diversity, its composition fluctuates at different stages of life and varies in healthy and sick persons [1]. The relationship between some gut flora and humans is commensal, of harmless co-existence, and mutualistic. The microbial composition of the gut microbiota also differs in different sections of the GIT. Very few species of bacteria are generally present in the stomach and small intestine, in comparison to the colon, which harbors the highest microbial population. Over 99% of the bacteria present in the gut are anaerobes. The dominant strains of bacteria isolated from the human gut were identified as belonging to five major phyla, i.e., Firmicutes, Bacteroidetes, Actinobacteria, Proteobacteria, and Verrucomicrobia [1,2].

An individual’s microbiota plays a vital role in the sustainability of health and the development of diseases [3,4]. Many factors induce changes in the composition and function of gut microbiota, such as an imbalanced diet, malnutrition, environmental factors, hygiene habits, immuno-compromised health conditions, short- or long-term usage of antibiotics, etc. [5]. A persistently disturbed microbiota might result in several ailments [6] and chronic diseases [7] (Table 1).

## 2. Influence of Probiotics and Functional Foods on the Gut Microbiota

Natural or processed foods that contain biologically active ingredients are termed functional foods. These, also known as nutraceuticals, can be defined as food containing additives that provide nutritional value with extra health benefits. Several reports established that the gut microbiota can be targeted and manipulated by suitable dietary means to prevent several temporary health issues and alleviate some diseases [8]. Researchers have confirmed that the gut microbiome can be improved by the intake of prebiotic supplements and through the consumption of functional food based on probiotics [9]. Probiotics and foods prepared with probiotics are generally considered safe. okProbiotic cultures have been widely used in food, medical treatments, animal feed, etc. They are easily available and accessible and are not expensive [10].

### 2.1. Description of Probiotics

The definition of probiotics according to the Food and Agriculture Organization/World Health Organization is “Live microorganisms which when administered in adequate amounts confer a health benefit on the host” [11,12]. Probiotic microorganisms are generally lactic acid bacteria (LAB), which are included under the “Generally Recognized As Safe (GRAS)” category by the US Food and Drug Administration (FDA) [13]. LAB belong to the phylum Firmicutes, class Bacilli, and order Lactobacillales, which includes over 50 genera placed in six families, (comprising *Lactobacillus*, *Levilactobacillus*, *Lacticaseibacillus*, *Limosilactobacillus*, *Lactococcus*, *Pediococcus*, *Enterococcus*, *Leuconostoc*, *Oenococcus*, *Streptococcus*, *Tetragenococcus*, *Aerococcus*, *Carnobacterium*, *Weissella*, *Alloiococcus*, *Symbiobacterium*, *and Vagococcus*) and more than 300 species [14,15]. Table 2 presents some microbial strains that are commonly used as probiotics.

### 2.2. Characteristics of Probiotics

The microbial strains that are widely used in the food fermentation industry are mostly LAB [18]. Their characteristics include the competitive ability to create a low pH due to acid production (lactic acid) and the production of primary and secondary antimicrobial metabolites, such as bacteriocins, hydrogen peroxide, diacetyl, and CO_2_. All these metabolites can play a role in the competition of LAB with other microorganisms during fermentation [19]. Due to their beneficial properties, LAB have been comprehensively explored in the food industry. For their beneficial properties, several strains of LAB are established as GRAS microorganisms in the U.S.A. and have been granted a “Qualified Presumption of Safety” (QPS) status in the E.U. *Lactococcus* and *Lactobacillus* have been given a GRAS status, the LAB genus *Streptococcus* and certain other species have been granted a GRAS/QPS status, whereas none of the species of the genus *Enterococcus* have been granted a GRAS/QPS status yet [22], since they probably include opportunistic pathogenic strains [23].

### 2.3. Use of Probiotics for Functional Foods

Foods containing an active live population of probiotics along with prebiotics are categorized as functional foods and have gained extensive popularity and acceptance in the health sector [20].

Global consumers have become aware of the importance of consuming a healthy diet to improve their health and sustain the quality of their lives [21]. Selected probiotic LAB strains exert established beneficial health effects such as the maintenance of the mutualistic intestinal microbiota by the inhibition of pathogens in the intestine. Studies have suggested probiotic strains as live cells are suitable starter cultures in functional food production. In addition, their metabolites may also be used as food additives and can be added directly to foods [12,24].

Through the consumption of functional food, probiotics stimulate a microbial balance in the gastrointestinal tract of the host. Extensive studies have established the beneficial effects of probiotics in the prevention of intestinal disorders, the protection against cancer, the activation of the immune function, the reduction of symptoms of irritable bowel syndrome (IBS), the lowering of cholesterol level, and in other processes, as summarized in Table 1 [20].

An active probiotic microbiota exerts several biological effects through diverse mechanisms. For example, for their survival in the GIT, they compete for nutrients and by doing so they prevent pathogenic microorganisms from adhering to the epithelial cells in the GIT. Secondarily, LAB produce antagonistic compounds like short-chain fatty acids (SCFA), bacteriocins, and organic acids, that inhibit pathogens’ growth and hinder the colonization of opportunistic microorganisms. Other health-promoting activities exerted by LAB are the regulation of the immune system by the stimulation of immunoglobulin production, the increase in the cytotoxicity of natural killer cells, and the modulation of cytokine secretion [25]. Therefore, considering the above-discussed benefits, the use of probiotics in functional foods has established a global business in the food industry.

## 3. Use of Probiotics in the Food Industry

LAB have been used as live cultures in artisanal food fermentation for a long time. They have been extensively used in the food industry as starter cultures due to the several desired characteristics linked to their metabolic activities that they impart to the final food products [26]. Their specific technological properties for producing specific metabolites are exploited in the food industry [27]. Fermentation by LAB add value to food and drink products by contributing texture, appearance, taste, aroma, and flavor [28,29].

A healthier version of “sourdough” bread has gained consumers’ interest. It is a slow-fermented bread prepared by a combination of LAB and yeast cultures. This bread is different from common bread because a live culture is used as a sourdough starter, which acts as a natural leavening agent. From a health perspective, this special bread has several properties compared to unfermented supermarket loaves. The naturally produced acids during slow and long fermentations help to break down the gluten present in the flour. This process makes this sourdough bread more digestible and easier for the body to absorb compared to unfermented normal bread [30,31]. Probiotic cultures present in Kefir grains have been studied for their beneficial effects in the production of “Functional Beverages” [9] and baking products [32,33]. The immobilization and encapsulation of LAB active cells in different systems have been studied to enhance their viability and hence their growth in a variety of conditions for the production of fermented food and drinks [34,35].

LAB have also proved their activity to control food-spoiling microorganisms. *Clostridium* spp. remain one of the main concerns causing economic, nutritional, and microbiological problems in the dairy industry. *Clostridium sporogenes* and *Cl. tepidium* strains with late blowing characteristics were detected in traditional cheese samples. They cause the swelling of cheese during aging. This phenomenon often affects hard-pressed cheeses and usually occurs from a few weeks to months of the aging process. Traditional Swiss-style cheeses are essentially meant to undergo late-blowing. This effect can be caused by several types of bacteria that are able to consume lactose, lactic acid, and remaining nutrient, and produce many different byproducts, including CO_2_, which causes gas bubble formation and holes in the mass of the cheese. Butyric acid fermentation is one of the frequent defects of hard or semi-hard cheeses, causing safety and economic problems [36]. *Clostridium* is an anaerobic Gram-positive spore-forming and gas-producing bacterium that is considered as the main agent causing late-blowing in cheeses [37]. To control *Clostridium* spp. in a variety of Turkish Kashar cheese, LAB strains were tested for their anti-clostridial activity. *L. plantarum* Y48 and *Lc. lactis* subsp. *lactis* PY91K were found to be effective in *in vitro* experiments, and then their dual effect as adjunct cultures was tested for the inhibition of *Clostridium* spp. in the production of Kashar cheese to prevent the undesirable late-blowing effect [38].

## 4. Use of Probiotics for Pharmaceutical Properties

LAB have proved their ability to synthesize many metabolites with pharmaceutical properties beneficial to health [39,40,41]. They also secrete exo-polysaccharides in the fermentation medium, which have been shown to have antidiabetic, antioxidant, and immunomodulatory properties. The peptides synthesized by LAB showed antimicrobial and anti-inflammatory activities, and β-galactosidases produced by LAB have found their application in improving lactose digestion [39].

### 4.1. Antimicrobial Properties

The antimicrobial properties of probiotic cultures may be due to their competition with foodborne pathogens for scavenging nutrients for their colonization in the GIT of the host. A variety of important compounds that they produce, such as organic acids (lactic, malic, and fumaric acid), hydrogen peroxide, exopolysaccharides, bacteriocins, and similar inhibitory substances, possess antagonistic activity against many undesirable and pathogenic microorganisms [40]. Bacteriocins are peptides produced by bacteria with antimicrobial activity, with either bacteriostatic or bactericidal activities against pathogens, that have not been found to harm the producing bacteriayes. These antimicrobial peptides are heat-stable and have a vast potential for their application as food preservatives and as antibiotics to treat multiple-drug resistant organisms [40]. The production of such antimicrobial compounds by Probiotics through their metabolic activities enhances the functional properties of probiotics; therefore, they could be beneficial for the prevention of foodborne pathogens and for relieving symptoms of some diseases associated with pathogens [41].

The disturbance of healthy gut microbes is a common condition due to the short- or long-term usage of antibiotics by patients. In such cases, the diets of patients after a prescribed course of antibiotics can be supplemented with probiotics [42]. Some populations of probiotic strains taken with food or drinks might colonize the gut permanently, while some are lost in the course of time [43]. Probiotics that stabilize themselves in the gut are understood to contribute beneficial effects to the host. They can improve the metabolic activities and enable a long-lasting adjustment of the indigenous microbiota yes [43]. Therefore, the improvement in the adhesion of bacterial cells in the gut is crucial for the effective colonization and the maintenance of probiotics. Based on their efficiencies, various probiotics are recommended for the prevention and alleviation of several diseases [44].

A few studies have confirmed that some organic compounds and functional natural ingredients can specifically improve the adhesion of bacterial strains or stimulate the expression of intestinal cell adhesion proteins. The contribution of exopolysaccharide (EPS) secreted by a LAB strain isolated from dairy *Lactobacillus paracasei* subsp. paracasei BGSJ2-8 was studied for its adhesion to intestinal epithelial cells and was shown to help decrease *Escherichia coli*’s association with Caco-2 cells. Researchers reported the presence of EPS on the surface of *Lactobacilli* could enhance the communication between bacterial cells and the intestinal epithelium, through the adhesion of probiotic cells necessary for their gut colonization [45]. Wang et al. reported that liposomes coated with bacterial *S-layer* proteins (isolated from *Lactobacillus helveticus*) significantly enhanced the adhesion of liposomes to the GIT [46]. A report confirmed the adhesion changes of *Lactobacillus* cultured in milk supplemented with lactophospholipin could boost the adhesion of *Lactobacillus* to Caco-2 cells. This biochemical activity required the expression of the genes *EF-TU* and *Cnb* related to lactobacillus adhesion [47].

*Lactobacillus plantarum* is a lactic acid bacterium found in animal and human mucosae, as well as in the nutritive-rich environments of several fermented food items [48]. EPS are important biological products produced by some LAB. In addition to their health benefits, EPS are well recognized for their shelf-life enhancement properties in the food and dairy industry, and hence, they are commercially applied in several products for their ability to enhance food’s technical functionality [49]. In addition, EPS support the adhesion of LAB to eukaryotic cells and the human gut to obtain nutrients [50]. EPS are associated with the formation of biofilms and a medium for linkage to surfaces. In biofilms, EPS also perform many essential roles such as separating essential cations, cellular recognition, and host–pathogen interactions [51].

### 4.2. Therapeutic Aspects

The health benefits offered by LAB are also nutritionally-therapeutic and include their role in vitamin production, allergies, and immunoregulation [52], the relief of lactose intolerance symptoms [53], the reduction in the risk of Crohn’s disease [54] and diabetes alleviation [55], or even have anti-cancer properties [56].

Antibiotic therapy is a common practice for the treatment of microbial infections, but as a result, the gut microbiota is disturbed, and in some patients this causes the initiation of diarrhea. Consumption of probiotic fermented foods with live LAB or commercially available probiotic preparations may prevent gastrointestinal disruption during and after antibiotic therapy by helping to re-establish the normal microbiota of the intestine [57,58,59].

### 4.3. Inflammatory Disease

The disruption in GIT microbiome also disrupts the physical and microbial barriers of the intestine, which affects the intestinal permeability and, in due course, may favor inflammation and systemic diseases [60]. IBD is often linked to a condition of dysbiosis accompanied by a shift towards a high accumulation of bacteria capable of managing oxidative stress, with a significant increase in bacteria of the Enterobacteriaceae family. A probiotic strain of *Lactobacillus gasseri* has been reported to exert anti-inflammatory effects in mouse colitis models, where it was able to maintain the integrity of the gut barrier. Hence, results suggested the protective role of this strain of probiotic against the progression of inflammatory intestinal diseases, such as IBD [61].

Studies have confirmed that EPS secreted by LAB have exclusive properties in modifying the gut microbiota [62]. EPS also act as a source of carbon, helping the growth and colonization of gut bacteria by feeding them nutrients [63]. The primary role of tight junction proteins claudin-1, ZO-1, and mucin-2 is the regulation of the intestinal barrier function, which prevents bacteria and toxins from entering the vascular system [64]. EPS isolated from LAB have shown the potential to act as prebiotics to promote the increase of probiotics, providing support for the adhesion of probiotics in the GIT and their long-term survival, necessary for their effective propagation. In a study, EPS were isolated from *L. plantarum*, and observations showed their effectiveness in enhancing the adhesion rate of *L. paracasei* cells to Caco-2 cells. Researchers claimed that previous works indicated that only a small number of prebiotics act as connectors between probiotics and GIT cells in the host, whereas most prebiotics do not influence the adhesion of probiotics [65,66,67]. Reports confirmed that EPS can enhance the adhesion of LAB of different species, and the adhesion rate was positively affected by the strength of EPS. This mechanism of action of EPS produced by LAB has a definite inhibitory effect on cancer cells [68].

Lipopolysaccharides (LPS), also often called endotoxins, are lipid-soluble outer-membrane components mainly secreted by Gram-negative bacteria. LPS levels have been found to be significantly increased in many studies of inflammatory diseases and diabetes [69,70,71]. LPS can penetrate through the intestinal epithelial cells and, after binding to chylomicrons, are transported to insulin-sensitive organs, causing insulin resistance and inflammation [72]. One of the main characteristics of type 2 diabetes is chronic inflammation; patients with this condition present excessive levels of inflammatory markers [73]. The levels of these pro-inflammatory elements were linked to those of LPS and free fatty aciyesds and are considered the important link between inflammation, obesity, insulin resistance, and type 2 diabetes. Reports have confirmed that the inflammation induced by LPS is one of the causes of dysfunction of pancreatic beta-cell [74]. Diabetic mouse models have shown acute inflammation and structural abnormalities in their tissues. Fatty liver disease steatosis was also shown in the diabetic group due to the excessive accumulation of lipid metabolites [73].

### 4.4. Diabetes Mellitus

The gut microbiota has also been indicated to be associated with the development of diabetes, probably through its role in regulating the immune response, because the abundance and composition of the gut microbiota vary with the quality of diet and imbalanced nutrition. Because of their exceptional advantages, probiotics have been extensively studied in T2D disease models. Disturbances in the gut microbiota can aggravate Type 2 Diabetes (T2D); however, the gut microbiota could also be affected in a patient with T2D. In a recently published report, the effect of exopolysaccharides synthesized by a strain of probiotic bacteria *Lactobacillus plantarum* on the adhesion of cells of another LAB, *Lactobacillus paracasei*, was studied [75].

EPS produced by *L. plantarum* was used in *in vivo* experiments. The results showed the adhesion of *L. paracasei* cells to Caco-2 cells was two-fold, and thereby, the cells of *L. paracasei* could maintain their propagation. The change in intestinal microbiota due to *L. plantarum* activities was beneficial in supporting the balance of desired strains of Bifidobacterium and Faecalibaculum. Additionally, their activities inhibited the colonization of other strains of bacteria involved in energy metabolism, such as Muribaculaceae, Firmicutes, and Lachnospiraceae. Researchers indicated that the correction in the microbiota improved the intestinal barrier, which was essential for the secretion of the gut hormones peptide YY and glucagon-like peptide-1 [76].

A report by Zhao et al. [75] indicated the combined function of EPS and a probiotic strain as symbiotic in alleviating T2D. By balancing the pro-inflammatory factors IL-6 and TNF-α with the anti-inflammatory factor IL-10, inflammation could be considerably reduced. Through the interactions between gut microorganisms and yestheir effect on the gut epithelial barrier, T2D can be controlled. The consumption of a choice of probiotics (presented in Table 3) by healthy consumers and patients is aimed to regulate the intestinal microbiota and could also be an effective accessory treatment for T2D and non-alcoholic fatty liver disease [77]. Various mechanisms are thought to contribute to the alleviation of T2D. The gut microbiota alters the micro-ecological structure of the host gut, reduces LPS-producing Gram-negative bacterial strains, and increases the population of SCFA-producing strains [78,79], facilitating farnesoid X receptor (FXR) signaling for the regulation of the bile acid metabolism [80], regulating the secretion of intestinal hormones peptide YY and glucagon-like peptide-1 [81], and more importantly, strengthening the intestinal barrier function, thus reducing the intestinal permeability [82,83]. T2D is a chronic disease that develops as a result of an unhealthy lifestyle, which can also disturb the microbiota, either due to therapy, or due to the consumption of an unhealthy diet. This can have an influence on the progression of T2D; therefore, it would be beneficial to take intervention measures in daily diets (Table 3) to restore the disturbed gut microbiota in patients with T2D [84].

### 4.5. Anti-Cancer Properties

With a better awareness of the role of the microbiome in the pathogenesis of cancer, the potential of microbiota-based therapeutics has become an increasingly researched topic in the treatment of cancer. Probiotics are microorganisms providing health benefits to the host by restoring or improving the gut microbiota when they are consumed in the required amount [12]. They exert many health-promoting effects, such as antioxidant activities, stimulation of the host immune system, and anticancer activity. Cell wall components of specific strains of *Kluyveromyces marxianus* and *Saccharomyces cerevisiae* var. *boulardii* have been reported to act as cancer chemo-preventive and antiproliferative and showed superoxide anion scavenging properties [85]. Probiotic cultures preventing the adherence of pathogens in the gut are considered to be live bio-therapeutics [86]. It is worthwhile to note that some compounds such as bioactive peptides in probiotics supernatants can contribute to health benefits through antioxidant and antitumor activities [87,88].

A high number of *Bacteroides massiliensis* was detected in samples of patients suffering from prostate cancer, suggesting the potential role of these bacteria in the expansion of prostate cancer [89]. In a study by Chung et al. [90], a *Bacteroides fragilis* toxin was shown to activate a pro-carcinogenic inflammatory pathway in colonic epithelial cells. The gut microbiome has also been shown to be involved in the carcinogenesis of colorectal cancer, with *Bacteroides fragilis*, *Fusobacterium nucleatum*, and *Peptostreptococcus anaerobius* being highlighted as potential players in its development [91]. A diet rich in whole grains and dietary fiber is associated with a lower risk of *F. nucleatum*-positive colorectal cancer, suggesting that the intestinal microbiome could be an important mediator in the interaction of diet and colorectal cancer [92,93].

*Saccharomyces boulardii*, a variety of *S. cerevisiae*, is used as a probiotic yeast in the food and drug industries. However, *S. boulardii* is an opportunistic pathogen, but the culture supernatant of *S. boulardii* contains different compounds with health benefits and without pathogenic and toxicity activities. The supernatant of this organism has been recommended for its health-promoting benefits. *S. boulardii* is commonly used as a therapeutic agent to prevent or treat diarrhea and other GI disorders in neonates and adults occasionally [94]. The effects of *S. boulardii* supernatant (SBS) on cell viability have been described, with the induction of apoptosis and the suppression of survivin gene expression in MCF-7 and MCF-7/MX cells,non-drug-resistant and multidrug-resistant breast cancer cells, respectively. SBS is suggested as a prospective anticancer drug to be administered in addition to standard treatments like surgery and chemotherapy to treat human breast carcinoma [95]. The overall evidence so far is weak, and research is still ongoing.

*Escherichia coli* Nissle 1917 is well studied as a versatile probiotic strain and has a long track record of safety in humans. Therefore, it has been used as a popular starting point for engineered therapeutic microbe efforts because of its compatibility with canonical genetic engineering techniques for bacteria [96]. This strain is used as a supplement for general gastrointestinal disorders and has also been evaluated for maintaining remission in ulcerative colitis in randomized control trials [97]. Studies have shown some favorable results, though with low efficacy, in the treatment of Inflammatory Bowel Disease [98]. Researchers have used *E. coli* Nissle 1917 as a cellular chassis for probiotic-associated therapeutic curli hybrids. Engineered *E. coli* Nissle 1917 was used for the delivery of matrix-tethered therapeutic domains to the gut [99].

## 5. Fecal Microbiota Transplantation

In this review, all cited published work mainly deals with scientific data on the influence of diets and supplements in controlling and restoring the disturbed gut microbiota and, consequently, its therapeutic effect. The introduction of probiotic bacteria in the GIT through the consumption of fermented food and drinks, as a source of nutrients and probiotics, is not expected to cause disturbances in the normal microbiota. We have not discussed Fecal Microbiota Transplantation (FMT), the process of transferring fecal bacteria and other microbes from a healthy individual into a sick individual. FMT has been suggested as an effective treatment for *Clostridioides difficile* infection causing acute diarrhea, where there is the concern that introducing probiotics using this process will delay the return to a normal microbiota. FMT has been widely accepted as an attempt to establish the microbiome’s pivotal role in gut dysbiosis-related disease models and as a new disease-altering therapy. Regardless of the potential beneficial results of FMT reported in various disease models, there is a discrepancy in the procedural agreement for performing FMT reported by different research groups. Even though there are many studies using FMT to test the causal links between the microbiome and diseases, a large number of variables of FMT procedures differ between studies, and there is no scientific agreement on a standard methodology [100].

## 6. Conclusions

In the last few decades, there has been a rise in the number of studies on the gut microbiome, and the focus of research has begun to move towards clinical and therapeutic studies to understand how the microbiome can influence human health and be effective in the alleviation of several diseases [101]. Nevertheless, the study of the gut microbiome is not without its drawbacks, and therefore, research needs to be continued to enhance our understanding of the microbiome to sustain and improve our health. Because of the diversity, variability, and complexity of the gut microbiota, the balance in its composition could be damaged by many factors at different stages of human life, as well as due to certain illnesses. Therefore, the modulation of interactions between microbial species, through the intervention of probiotics and with the use of EPS produced by LAB as prebiotics, could be an important strategy to sustain good health and alleviate several diseases.

## Figures and Tables

**Table 1:** Role of the Gut Microbiota in Health and Disease [1,2,3,4,5,6,7] (table drawn by us, information collated from several sources).

Beneficial Effects	Damaging Effects
An important role in the digestion	Gastrointestinal disorders, Increased risk of Diarrhea
Supply of nutrients by the synthesis of Vitamins and Antioxidants	Metabolic Disorders
Degradation of Xenobiotics	Kidney disease
Building and stimulating the Immune system by reducing inflammation in the gut	Colon cancer, Irritable Bowel Syndrome (IBS), Inflammatory Bowel Disease (IBD)
Development of Cognitive abilities, Gut–brain axis	A decline in Cognitive abilities
Improved lipid metabolism	Liver inflammation
Shielding against pathogens, protection of epithelial cells of the gut	Obesity
Inactivation of invader and opportunistic microbes	Onset and progression of infectious disease
Insulin sensitivity	Insulin resistance, Diabetes mellitus
Prevention of cardiovascular diseases	Increased risk of CVD

**Table 2:** Microorganisms used as probiotics in food fermentation and oral supplements [16,17,18,19,20,21].

Strains Used for the Production of Fermented Food Products	Individually Microencapsulated Freeze-Dried Strains in Commercial Supplements (Capsules)
*Lactobacillus acidophilus both columns have no relation* *L. sporogenes * *L. paracasei * *Lactiplantibacillus plantarum * *Lacticaseibacillus rhamnosus * *Limosilactobacillus reuteri * *Limosilactobacillus fermentum* *Levilactobacillus brevis * *Lacticaseibacillus casei * *Lactococcus lactis subsp. cremoris * *Streptococcus salivarius* *Kefir grains* mixture of LAB and yeast	*Bacillus subtilis* *Bifidobacterium bifidum* *B. breve* *B. infantis* *B. longum* *Lactobacillus acidophilus* *L. delbrueckii subsp. bulgaricus* *L. casei* *L. plantarum* *L. rhamnosus* *L. helveticus* *L. salivarius* *Lactococcus lactis subsp. lactis* *Streptococcus thermophilus*

(Ref information collated from several sources).

**Table 3:** Sources of probiotics to influence the gut microbiota for use according to consumers’ personal choice [9,10,18,20,26,27,28,29,30,31,32,33].

Traditional Fermented Food/Drink Products	Commercial Food/Drink Products Available in Supermarkets	Commercial Supplements
Sauerkraut, Fermented white cabbage	*SKYR*—Icelandic dairy product	By 2023, probiotic supplement sales are projected to exceed 64 billion dollars
Kimchi, Fermented vegetables	Natural Yoghurt, milk fermented by lactic acid bacteria	Sold in health shops Several brands (claiming a potency from 2 to 25 Billion CFUs)
Tempeh, Fermented Soybean product	Kefir, fermented milk Functional-beverage, Several fruit-flavored varieties	Online sale by several companies
Miso, Fermented soybeans with Koji fungus	Smoothies, Blend of fruits, vegetables with probiotic-rich yogurt	Capsules Probiotic Ultimate Flora
Kombucha, Fermented black or green tea	Sourdough bread	High-dose probiotic drinks containing *Lactobacillus paracasei*, *L. casei*, *L. fermentium*
Umeboshi, Japanese fermented plums	Cottage cheese variety fermented with active LAB cultures	Capsules containing a multi-strain probiotic blend
Utonga-kupsu, fermented fish	Sour cream with live active LAB cultures	Capsules with *Lactobacillus rhamnosus* GG strain
Natto, a Japanese fermented soybean product	Variety of cheeses, only if labeled “live cultures” or “active cultures”	Delayed-release capsules with a blend of Prebiotics + Probiotics
Traditional preparation of Buttermilk, Kefir grains, fermented milk, natural yogurts	Unpasteurized pickled Vegetables	Bio-Kult with 14 probiotic strains, incl. *Lactobacillus acidophilus*, *Streptococcus thermophilus*, *Bifidobacterium longum*
